# Development of Water Retentive and Thermal Resistant Cement Concrete and Cooling Effects Evaluation

**DOI:** 10.3390/ma14206141

**Published:** 2021-10-16

**Authors:** Xiaowei Wang, Xinyu Hu, Xiaoping Ji, Bo Chen, Hongqing Chen

**Affiliations:** 1 School of Civil Engineering, Xi’an University of Architecture & Technology, Xi’an 710055, China; 2 Key Laboratory of Special Area Highway Engineering of Ministry of Education, Chang’an University, Xi’an 710064, China; xinyu_hu2021@163.com (X.H.); jixp@163.com (X.J.); 3 No. 1 Municipal Administration Institute, Xianyang Planning & Design Institute, Xianyang 712021, China; song_quan@163.com; 4 CCCC-SHEC Third Highway Engineering Co., Ltd., Xi’an 710016, China; suyunfei030@163.com

**Keywords:** urban heat island effects, water retentive material, water retentive and thermal resistant cement concrete, pavement temperature reduction

## Abstract

The high pavement temperature plays an important role in the development of urban heat island (UHI) in summer. The objective of this study was to develop water retentive and thermal resistant cement concrete (WTCC) to enhance the pavement cooling effects. The WTCC was prepared by combining a water retentive material and a high aluminum refractory aggregate (RA) with porous cement concrete (PCC). Water retention capacity test, fluidity test, and compressive strength test were used to determine the composition ratio of the water retentive material. Mechanical performance and cooling effects of WTCC were evaluated by compressive and flexural strength tests and temperature monitoring test. The mass ratios of fly ash, silica fume, cement, and water in the water retentive material were determined as 65:35:15:63.9. The compressive strength and the flexural strength of WTCC after 28 days curing were 30.4 MPa and 4.6 MPa, respectively. Compared with stone mastic asphalt (SMA) mixture, PCC, and water retentive cement concrete (WCC), surface temperature of WTCC decreased by 11.4 °C, 5.5 °C, and 4.1 °C, respectively, and the internal temperatures of WTCC decreased by 10.3 °C, 6.1 °C, and 4.6 °C, respectively. The water retentive material has benefits of strength improvements and temperature reduction for WTCC. Based on the results, WTCC proved to have superior cooling effects and the potential to efficiently mitigate the UHI effects and be used in medium traffic roads.

## 1. Introduction

With global warming and fast urbanization, temperatures in metropolitan areas are significantly greater than the surrounding suburban areas in summer, and this phenomenon is named urban heat island (UHI) [1,2]. Studies demonstrated that pavement contributes highly to the development of UHI because the pavement temperature is very high in summer, and the pavement area percentage in urban areas may be up to 40% [3,4,5]. Especially, asphalt pavements have a more significant influence on the UHI effects because the heat absorptivity and the temperature of asphalt pavement are higher than other pavements [6]. The high temperature not only causes pavement distress [7] but also aggravates the UHI effects [1,8]. Therefore, developing new pavement technologies to reduce the pavement temperature has a great significance for pavement durability and sustainability of the environment.

There are mainly four ways to reduce the pavement temperature: (a) increasing the albedo of pavement surface; (b) decreasing the thermal conductivity of pavement materials; (c) utilizing water retentive pavements; and (d) using phase change materials. Increasing the albedo of pavement, known as reflective pavement, has been used for a long time to reduce the pavement temperature [1,9]. In the eastern San Francisco Bay Area of California, the albedo and the temperature of different asphalt pavements were measured. Results indicated that the temperature and the albedos correlated well with each other. The albedo of chip-seal was about 70% of the aggregates, resulting in a surface temperature reduction of about 9.0 K compared with the traditional asphalt pavements [10]. Furthermore, coloring additives together with proper aggregates were used to increase the albedo of the pavements, and surface temperature decreased about 12 K compared to weathered asphalt [11]. In general, reflective pavements are mainly carried out using the following strategies: (a) use of white high reflective paints on the surface of the pavement [11,12]; (b) use of infrared reflective colored paints on the surface of the pavement [3,13]; (c) use of heat reflecting paints to cover aggregates [9]; and (d) use of color changing paints on the surface of the pavement [14]. The durability of the reflective pavements is the main concern in the practices.

Apart from the reflective pavements, permeable pavements and thermal resistant pavements are other pavement technologies available to reduce the pavement temperature through decreasing the thermal conductivity of pavement materials. The permeable pavements are paved by porous cement concrete or porous asphalt mixtures, which have a high air void (AV) content and lower thermal conductivity than traditional dense-graded asphalt mixtures [15,16]. Studies reported that the insulation properties of permeable pavements contributed to a temperature reduction [17,18]. Alternative aggregates with a lower thermal conductivity incorporated into asphalt mixtures have been applied to reduce pavement temperature. The thermal conductivity of ceramic, floating beads, and refractory gravel is lower than ordinary aggregates. Pavement temperature reduction was observed when the ordinary aggregates were substituted by these lower thermal conductivity aggregates [2,6]. In addition, water retentive pavements were used to decrease the pavement temperature by dissipating the heat through water evaporation [8]. Water absorption capability and water retention capacity of sintered ceramic pervious brick (CB), pervious concrete brick (PB), and open-graded pervious concrete (PC) were investigated. Results revealed that CB and PB could reduce the surface temperature by up to 20 °C and 12 °C with cooling periods of 16 h and 12 h, respectively [19]. To extend the evaporative cooling period of water retentive pavements, water retentive materials that use highly absorptive filler, such as sepiolite, fly ash, or blast furnace slag, were added into the pore structures of pervious pavements [5,20,21]. Super absorbent polymer (SAP) was added into porous asphalt mixtures, showing a significant cooling effect [22]. Recently, phase change materials incorporated with a lightweight aggregate were used in asphalt mixtures to reduce pavement surface temperature [23,24]. Surface temperature decreased about 4 °C under a test temperature range between 30 °C and 60 °C [24]. The phase change materials also were used in a cement mortar to accumulate heat [25].

**Figure 1:**
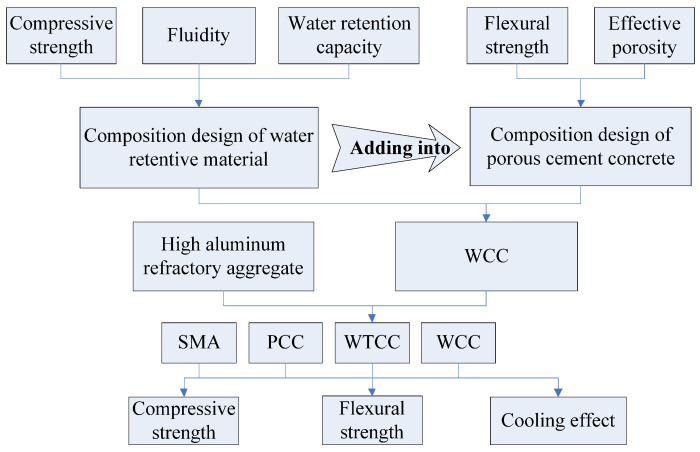
Schematic development of WTCC and test program.

In this study, a water retentive material was designed and combined with a thermal resistant aggregate and porous cement concrete (PCC) to develop a water retentive and thermal resistant cement concrete (WTCC). With lower thermal conductivity PCC and water evaporation of a water retentive material, the WTCC may have the potential to enhance the pavement cooling effects. The mechanical performance and the cooling effects of WTCC were also evaluated and compared with stone mastic asphalt mixtures (SMA), PCC, and water retentive cement concrete (WCC). The schematic development of WTCC and the test program is shown in Figure 1.

## 2. Materials and Sample Preparation

The main raw materials for preparing WTCC included thermal resistant aggregate, cement, silica fume, fly ash, and water. In addition, SMA, PCC, and WCC were also prepared and compared with WTCC. A summary of the main composition of these mixtures is shown in Table 1. A water retentive material was designed for preparing the WCC and the WTCC.

Styrene-butadiene-styrene (SBS) modified asphalt was used for SMA, while cement graded 42.5 was used for PCC, WCC, and WTCC. The properties of SBS modified asphalt and cement are listed in Table 2 and Table 3, respectively. Two types of coarse aggregates—serpentinite aggregate (SA) and thermal resistant aggregate of high aluminum refractory aggregate (RA)—were used, and the properties of the coarse aggregates are summarized in Table 4. SA with different sizes was used for SMA, PCC, and WCC, while RA with a uniform size of 5–10 mm was used for WTCC. RA was produced by Xi’an Hua Rong Refractories Co., Ltd. The thermal conductivity of RA was 0.5 W·(m·K)^−1^, which is significantly lower than the ordinary aggregates of SA.

### 2.1. Water Retentive Material

Water retentive material was designed to improve the absorption and the retention capacities of WCC and WTCC. The water retentive material was composed of fly ash, cement, and silica fume. Fly ash is a porous material with strong water absorption and retention capacity. Silica fume is a non-crystalline polymorph spherical particle with smooth surface, large specific surface area, and high activity. In the water retentive material, silica fume mainly acted as a lubricant to improve the fluidity, and cement provided the strength. The chemical compositions of fly ash and silica fume are shown in Table 5.

To prepare the water retentive material, first, cement, fly ash, and silica fume were weighted according to the composition ratio. Next, all the materials were mixed together with water, and the mixture was stirred until uniform consistency slurry was formed. The water retentive material should have good workability in order to easily infiltrate the pore structure of PCC as well as good water retention capacity to store water. The composition ratio of the water retentive material was determined based on water retention capacity (W_R_), fluidity (F_L_), and compressive strength and is discussed in the following section.

### 2.2. Stone Mastic Asphalt (SMA)

**Figure 2:**
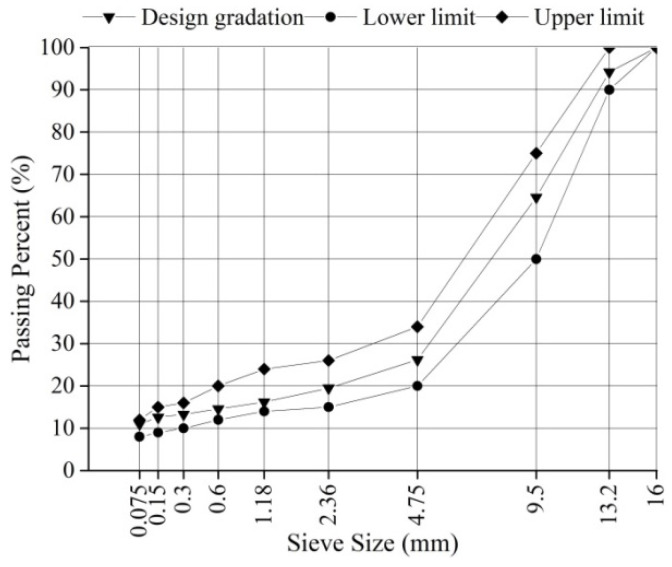
Gradation of SMA-13.

SMA with a nominal maximum aggregate size (NMAS) of 13.2 mm (SMA-13) was investigated and used as the surface layer of SMA pavements. SMA-13 was designed based on the Marshall mixture design method [26]. The asphalt content was 6.1%, and the AV content was 3.5%. The gradation of SMA-13 is shown in Figure 2. A rolling compaction machine was used to prepare the specimen with a length of 300 mm, a width of 300 mm, and a height of 50 mm. Another dense graded asphalt mixture with a NMAS of 19 mm (AC-20) was compacted with the same dimensions. AC-20 was the bottom layer of SMA pavement. The SMA-13 specimen was bonded with the AC-20 specimen using emulsified asphalt to simulate the SMA pavement structure. Thus, the total height of the SMA specimen was 100 mm.

### 2.3. Porous Cement Concrete (PCC)

PCC is the matrix for preparing WCC and WTCC and providing strength for them. Two types of PCC were prepared in this study. One was PCC prepared by SA (PCC-SA), and the other was PCC prepared by RA (PCC-RA). WCC was prepared by pouring the water retentive material into PCC-SA, and WTCC was prepared by pouring the water retentive material into PCC-RA. PCC-SA and PCC-RA were designed based on target valid interconnected AV content (AV_valid_), workability, and flexural strength. The target AV_valid_ of the PCC was 18–22%, and the AV_valid_ was calculated based on the Equation (1). The specimens used in the AV_valid_ measurement were cubic specimens with lengths, widths, and heights of 150 mm after 7 days of curing. The compositions of the PCC-SA and the PCC-RA are shown in Table 6.

(1) $$AV_{\mathrm{valid}}=(1-\frac{m_{\mathrm{water}}-m_{\mathrm{dry}}}{V\cdot \rho_{w}})\times 100\%$$

where m_water_ is saturated specimen mass in water, g; m_dry_ is mass of specimen in air, g; V is the volume of specimens, cm^3^; and ρ_w_ is density of water, g/cm^3^.

## 3. Test Methods

Water retention capacity test, fluidity test, and compressive strength test were used to evaluate W_R_, F_L_, and compressive strength of the water retentive material. In addition, the compressive strength test, the flexural strength test, and the temperature monitoring test were utilized to evaluate the mechanical performance and the cooling effects of WTCC.

### 3.1. Water Retention Capacity Test

The water retentive material should have good W_R_ to store as much as water as possible. The greater W_R_ is, the longer the evaporation time lasts. W_R_ was determined by the following steps:

1. Water retentive material was prepared according to the composition ratio. A cylindrical specimen with a height of 5 cm and diameter of 5 cm was fabricated.
2. The specimens were cured at a constant temperature of 20 ± 2 °C and a humidity of 95% for seven days.
3. Specimens were dried at a constant temperature of 105 ± 2 °C for 24 h, and the original mass of the specimens (m_0_) was measured.
4. Specimens were immersed into water until the mass was constant. The mass of the saturated specimen was measured as m_1_. The W_R_ is calculated by the Equation (2).
  (2) $$W_{R}=\frac{m_{1}-m_{0}}{V}\times 100\%$$
  where V is the volume of specimens, cm^3^.

### 3.2. Fluidity Test

The F_L_ was determined via flow cone method according to JTG 3420-2020 [27]. A volume of 1725 mL ± 5 mL of water retentive material was poured into a flow cone by opening the outlet and allowing the water retentive material to free flow out from the flow cone. The flowing time was recorded as the F_L_. The greater F_L_ was, the better the fluidity was.

### 3.3. Compressive Strength and Flexural Strength Test

For the water retentive material, compressive strength test was performed on cylindrical specimens with a height of 50 mm and diameter of 50 mm. The specimens were cured at a temperature of 20 ± 2°C and a humidity of 95% for seven days. A loading rate of 5 mm/min was applied until the specimens failed. The compressive strength (R_c_) of the water retentive material was calculated based on Equation (3).

(3) $$R_{c}=\frac{P}{A}$$

where P is the peak force, kN; and A is the area of upper surface of specimen, mm^2^.

For PCC, WTCC, and WCC, both compressive strength and flexural strength were evaluated. Prismatic specimens with a height of 150 mm, a width of 150 mm, and a length of 550 mm were prepared for the measurement of flexural strength. Four point bending test was used to measure the flexural strength. Cubic specimens with height, width, and length of 150 mm were prepared for the compressive strength test [27]. The compressive strength was calculated based on the Equation (3), and the flexural strength (F_f_) was calculated based on the Equation (4). The compressive strength and the flexural strength of the specimens after 7 days, 28 days, and 90 days curing were evaluated.

(4) $$F_{f}=\frac{PL}{bh^{2}}$$

where L is the length between two fixing supports, mm; and b and h are width and height of specimen, mm.

### 3.4. Temperature Monitoring Test

Temperature monitoring test was designed to evaluate the cooling effects of SMA, PCC, WCC, and WTCC. The specimens of PCC, WCC, and WTCC used in the temperature monitoring test had a length of 300 mm, a width of 300 mm, and a height of 100 mm. Vibration compaction was used to prepare the specimen of PCC, WCC, and WTCC. Before compaction, one temperature sensor was embedded at the depth of 50 mm, and another temperature sensor was embedded at the surface of the center of each specimen.

**Figure 3:**
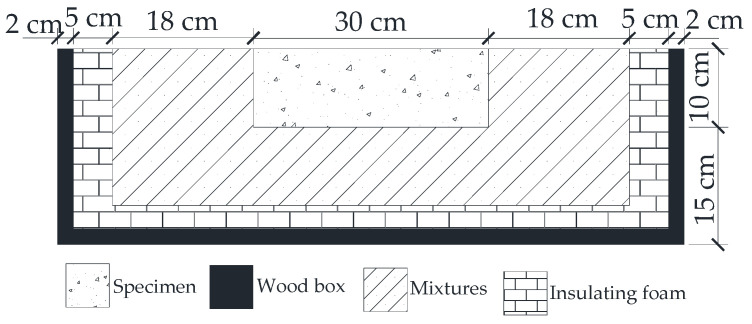
Schemes of testing box.

In actual pavement, the heat only can be transferred from the surface of the pavement. To simulate the field condition, a testing box was designed to avoid the heat transferring from the bottom and the side of the specimen. As shown in Figure 3, the testing box was a wood box filled with insulating foam and tested mixtures. The thickness of the insulating foam was 5 cm. The bottom and the side of the specimens were wrapped by the same mixtures with the tested specimens. 

A multi-channel automatic temperature recorder, produced by Hang Zhou Ze Da Instrument Co., Ltd., (Hangzhou, China) was used in the temperature monitoring test. The measuring accuracy was 0.1 °C, and the temperature was recorded every 10 min. Before testing, a mass of 500 mL water was sprinkled on the surface of the specimens to simulate the rainfall. Temperature sensors were connected to the multi-channel automatic temperature recorder. To evaluate the field cooling effects, one day with maximum and minimum air temperatures of 35 °C and 19 °C in summer was selected to perform the temperature monitoring test.

## 4. Results and Discussion

### 4.1. Design of Water Retentive Material

The composition ratio of fly ash, cement, silica fume, and water in the water retentive material was determined based on W_R_, F_L_, and compressive strength. The cement not only provided strength for the water retentive material but also prevented fly ash and silica fume from losing the water retentive material. However, excessive cement may clog the air voids and had a negative effect on the absorption capacity. Based on common sense and practices, the maximum percentage of cement is recommended as 15% of the total mass of fly ash and silica fume [28]. In addition, W_R_ greater than 0.25 g/cm^3^, F_L_ between 15 and 20 s, and compressive strength greater than 2.0 MPa are generally required to ensure workability and strength for the water retentive material [28].

**Figure 4:**
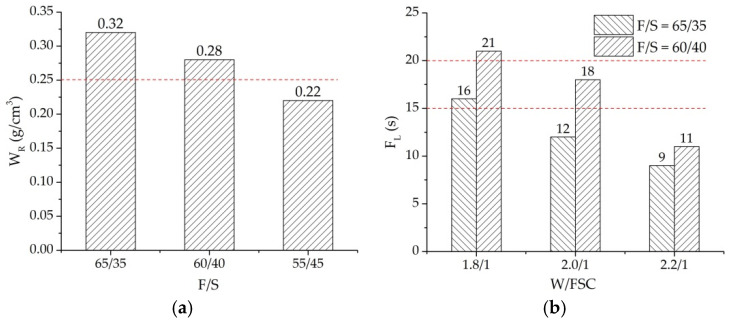
W_R_ and F_L_: (**a**) W_R_ under different F/S; (**b**) F_L_ under different W/FSC.

**Figure 5:**
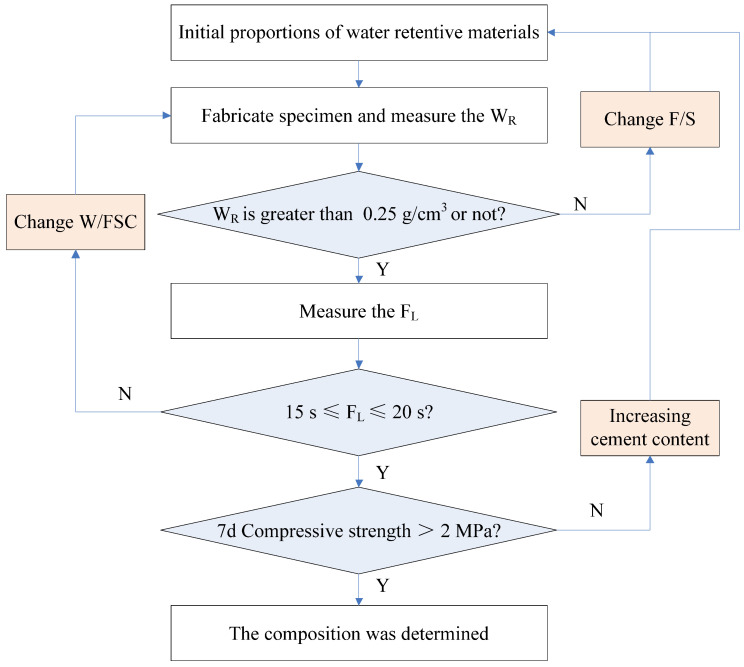
Design procedure of water retentive material.

The mass ratio of fly ash to silica fume (F/S) was firstly determined. Three F/Ss of 65/35, 60/40, and 55/45 were investigated by the water retention capacity test. Figure 4a shows the W_R_ under different F/S. W_R_ decreased with the decrement of F/S. Results indicate that increasing the percentage of fly ash can increase the W_R_ of the water retentive material. When the F/S was greater than 60/40, the W_R_ could satisfy the requirement of being greater than 0.25 g/cm^3^. Secondly, the mass of water to the total mass of fly ash, silica fume, and cement (W/FSC) was determined based on the F_L_. The fluidities of three W/FSCs of 1.8/1, 2.0/1, and 2.2/1 were evaluated. Figure 4b presents the F_L_ of different W/FSCs under the F/Ss of 65/35 and 60/40. The F_L_ decreased with the increasing W/FSC. Under the same W/FSC, F_L_ under F/S of 60/40 was greater than F/S of 65/35, demonstrating that silica fume had a positive influence on the fluidity of the water retentive material. Under the F/S of 65/35 with W/FSC of 1.8/1 and F/S of 60/40 with W/FSC of 2.0/1, F_L_ satisfies the requirement of between 15 and 20 s. Compared with W/FSC of 2.0/1, the water retentive material under W/FSC of 1.8/1 with less water had better durability because water had a negative influence on the durability. Thus, the composition of the water retentive material was determined as F/S of 65/35, cement mass of 15% of the total mass of fly ash and silica fume, and W/FSC of 1.8/1. In other words, the mass ratio of fly ash, silica fume, cement, and water was 65:35:15:63.9. Finally, the compressive strength test was performed, and the compressive strength was 2.7 MPa, which is greater than 2.0 MPa. The design procedure of water retentive material is summarized in Figure 5.

### 4.2. Preparation of WTCC

The WTCC was prepared by pouring water retentive material into PCC-RA, while WCC was prepared by pouring water retentive material into PCC-SA after 7 days of curing. The flexural strengths of PCC-SA and PCC-RA after 28 days of curing were 4.8 MPa and 4.3 MPa, respectively. The flexural strength of RCC-RA was smaller than RCC-SA because the strength of RA was smaller than SA. Based on the specification of design of highway cement concrete pavement of China, the minimum flexural strength of 4.5 MPa is specified for medium traffic roads, and the minimum flexural strength of 4.0 MPa is specified for light traffic roads [29]. Therefore, PCC-SA could be used for the medium traffic roads, while PCC-RA only could be used for the light traffic roads.

The preparation of WCC and WTCC was based on the following steps: (1) Dust was removed from the surface of the specimens, and some water was sprayed on the surface of the specimens. (2) We placed the specimens on a vibration table and poured the water retentive material on the surface of specimen. The surface of the specimen was covered by a geotextile. (3) We turned on the vibration table until the water retentive material could not infiltrate the specimens. (4) The water retentive material that remained on the surface was removed, and the specimens were cured under the temperature of 20 ± 2 °C and the humidity of 95% for 28 days.

### 4.3. Mechanical Performance of WTCC

**Figure 6:**
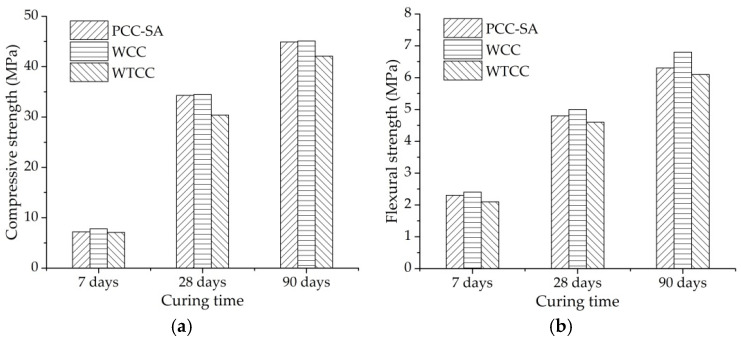
Compressive and flexural strength of different pavements: (**a**) compressive strength; (**b**) flexural strength.

Figure 6a,b show the compressive strength and flexural strength of different pavements after 7 days, 28 days, and 90 days curing, respectively. The compressive strength and flexural strength of WTCC at 28 days is 30.4 MPa and 4.6 MPa, respectively, which satisfies the requirement of medium traffic roads. Even though the WTCC used in the square, bicycle lane, and sidewalk is more appropriate, the WTCC has a potential used in the medium traffic roads based on the mechanical performance.

The compressive strength and the flexural strength increased with the curing time. The flexural strength of WCC was greater than PCC-SA, and the flexural strength of WTCC was greater than PCC-RA, indicating that the water retentive material functionally improved the flexural strength. After the water retentive material was added into the PCC-SA and the PCC-RA, the pore structures were filled with water retentive materials, leading to AV content decrement. The strength increased with the decrement of AV content, and the self-strength of the water retentive material could also improve the strength of the specimen. In addition, compared with PCC and WTCC, both compressive strength and flexural strength of WCC were the largest. It also demonstrates that the water retentive material and the higher strength of SA had contributions to the strength of the specimen.

### 4.4. Cooling Effects of WTCC

**Figure 7:**
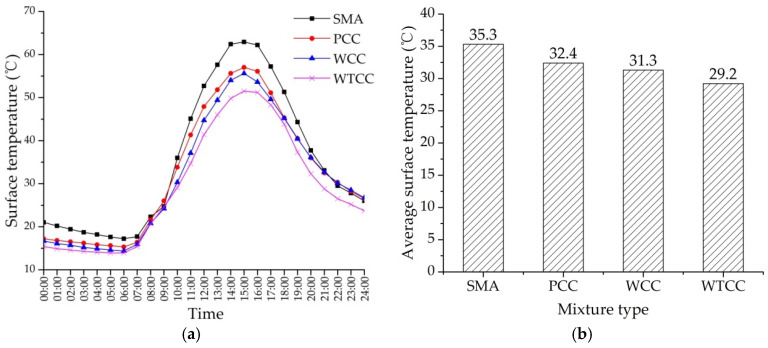
Surface temperature under field air temperature: (**a**) temperature variation curve; (**b**) average surface temperature.

**Figure 8:**
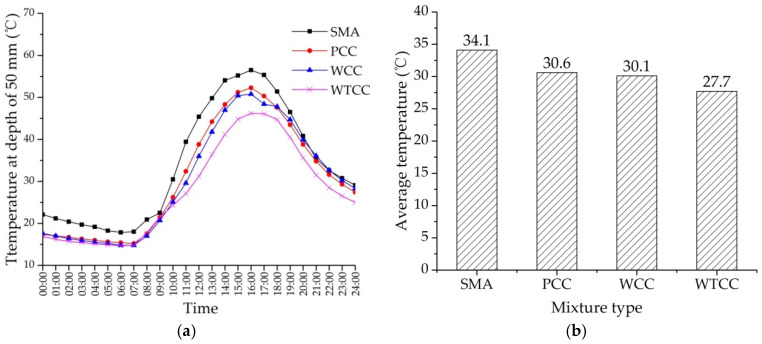
Temperature at the depth of 50 mm under the field air temperature: (**a**) temperature variation curve; (**b**) average temperature.

Figure 7 shows the surface temperature of four specimens under the field air temperature. As shown in Figure 7a, the temperature changes showed a consistency with the air temperature. At the start of the test, the temperature of cement concrete pavement was lower than SMA due to a lower heat absorptivity of cement concrete than asphalt mixtures. The highest surface temperature appeared at about 15:00, and the surface temperature of SMA was the highest. The highest surface temperatures of SMA, PCC, WCC, and WTCC were 62.9 °C, 57.0 °C, 55.6 °C, and 51.5 °C, respectively. Compared with SMA, PCC, and WCC, the highest surface temperatures of WTCC decreased by 11.4 °C, 5.5 °C, and 4.1 °C, respectively. Figure 7b shows the average surface temperature of the four mixtures. Compared with SMA, PCC, and WCC, the average surface temperatures of WTCC decreased by 2.9 °C, 4.0 °C, and 6.1 °C, respectively. Figure 8a shows the temperature distribution at the depth of 50 mm under the field air temperature. The temperature at the depth of 50 mm of SMA was also the highest. Compared with SMA, PCC, and WCC, the highest temperatures at the depth of 50 mm of WTCC decreased by 10.3 °C, 6.1 °C, and 4.6 °C, respectively. Figure 8b shows the average of the temperature at the depth of 50 mm. Compared with SMA, PCC, and WCC, the average surface temperatures of WTCC decreased by 3.5 °C, 4.0 °C, and 6.4 °C, respectively. A significant temperature reduction was found in WTCC.

The temperature of WCC was slightly smaller than PCC due to the heat dissipation through water evaporation of the water retentive material. However, as shown in Figure 7 and Figure 8, the temperature of WCC approached the same as the temperature of PCC after 18:00. Water in the water retentive material was consumed during continuous evaporation. When the water was exhausted, temperature reduction through water evaporation failed. Therefore, it is suggested to supplement water for pavement at 12:00 to prolong and enhance the cooling effects. This work can be done by incorporating it in the cleaning work of pavements. Because WTCC uses thermal resistant aggregates, the temperature of WTCC was still the lowest, even when the water in the water retentive material was exhausted.

Based on the results, WTCC presents a great cooling effect via the combination of porous cement concrete, a thermal resistant aggregate, and water retentive material. WTCC has potential to efficiently mitigate the UHI effects and be used in medium traffic roads.

## 5. Conclusions

In this study, water retentive material and WTCC were developed to alleviate the UHI effects. The mechanical performance and the cooling effects of WTCC were evaluated and compared with SMA, PCC, and WCC. Several conclusions could be obtained from the study.

(1) Based on water retention capacity, fluidity, and compressive strength, the composition ratio of the water retentive material was determined. The mass ratios of fly ash, silica fume, cement, and water were determined as 65:35:15:63.9.
(2) WTCC was prepared by pouring the water retentive material into PCC incorporating a thermal resistant aggregate. Even though the WTCCs used in the square, the bicycle lane, and the sidewalk were more appropriate, the WTCC has potential to be used in medium traffic roads based on the mechanical performance.
(3) The water retentive material has benefits of improving the strength of pavements and temperature reduction. The freeze–thaw resistance of WTCC will be further evaluated in future tests.
(4) Compared with SMA, PCC, and WCC, surface temperatures of WTCC decreased by 11.4 °C, 5.5 °C, and 4.1 °C, respectively, and internal temperatures decreased by 10.3 °C, 6.1 °C, and 4.6 °C, respectively. Results demonstrate that WTCC has superior cooling effects due to the lower thermal conductivity of porous cement concrete, the thermal resistant aggregate, and the water retentive material. WTCC can be used to efficiently to mitigate UHI effects.

## Figures and Tables

**Table 1:** Brief of the composition of different mixtures.

Mixture Type	Binder	Aggregate	Water Retentive Material
SMA	SBS modified asphalt	Serpentinite	No
PCC	Cement	Serpentinite	No
WCC	Cement	Serpentinite	Yes
WTCC	Cement	High aluminum refractory aggregate	Yes

**Table 2:** Properties of SBS modified asphalt.

Properties	Value
Penetration (25 °C, 100 g, 5 s) (0.1 mm)	66.2
Ductility (5 cm/min, 5 °C) (cm)	36.5
Kinematic viscosity (135 °C) (Pa·s)	1.965
Softening point (°C)	74.9
Elastic recovery (%)	99.0

**Table 3:** Properties of cement.

Properties		Value
Specific surface area (Blaine method) (m^2^·kg^−1^)		362
Normal consistency (%)		28.0
Setting time (min)	Initial setting time	230
	Final setting time	295
Flexural strength (MPa)	3 days	4.2
	28 days	7.9
Compressive strength (MPa)	3 days	20.1
	28 days	44.5

**Table 4:** Properties of coarse aggregates.

Properties	SA	RA	Limits
Crushed value (%)	6.8	26.8	≤30
Los Angeles abrasion loss (%)	5.6	28.4	≤35
Apparent specific gravity (g·cm^−3^)	2.964	2.830	≥2.45
Bulk specific gravity (g·cm^−3^)	2.896	2.732	-
Water absorption (%)	0.71	5.89	≤3.0
Thermal conductivity (W·(m·K)^−1^)	2.2	0.5	-

**Table 5:** Chemical compositions of fly ash and silica fume.

Elements	Fly Ash	Silica Fume
SiO_2_ (%)	38.29	85.6
Al_2_O_3_ (%)	22.83	0.81
Fe_2_O_3_ (%)	20.15	0.9
CaO (%)	1.18	0.3
MgO (%)	7.76	0.7
Ignition Loss (%)	15.2	1.0
Na_2_O (%)	/	1.3
Specific surface area (m^2^/g)	1.7	20.8

**Table 6:** Compositions of PCC-SA and PCC-RA.

Type	Materials Mass in One Cubic Meter/kg				AV_valid_ (%)
	Cement	Silica Fume	Aggregate	Water	
PCC-SA	266	30	1427	107	19.5
PCC-RA	266	30	1395	121	21.4

## Data Availability

The data presented in this study are available on request from the correspondence author.
